# Image augmentation and automated measurement of endotracheal-tube-to-carina distance on chest radiographs in intensive care unit using a deep learning model with external validation

**DOI:** 10.1186/s13054-023-04320-0

**Published:** 2023-01-25

**Authors:** Matthieu Oliver, Amélie Renou, Nicolas Allou, Lucas Moscatelli, Cyril Ferdynus, Jerôme Allyn

**Affiliations:** 1 Methodological Support Unit, Reunion University Hospital, Saint-Denis, France; 2 Intensive Care Unit, Reunion University Hospital, Saint-Denis, France; 3 Clinical Informatics Department, Reunion University Hospital, Saint-Denis, France; 4 Radiology, Reunion University Hospital, Saint-Denis, France; 5 Clinical Research Department, INSERM CIC 1410, F-97410 Saint-Pierre, France

**Keywords:** Intensive care unit, Chest radiograph, RetinaNet, Deep learning, Image processing

## Abstract

**Background:**

Chest radiographs are routinely performed in intensive care unit (ICU) to confirm the correct position of an endotracheal tube (ETT) relative to the carina. However, their interpretation is often challenging and requires substantial time and expertise. The aim of this study was to propose an externally validated deep learning model with uncertainty quantification and image segmentation for the automated assessment of ETT placement on ICU chest radiographs.

**Methods:**

The CarinaNet model was constructed by applying transfer learning to the RetinaNet model using an internal dataset of ICU chest radiographs. The accuracy of the model in predicting the position of the ETT tip and carina was externally validated using a dataset of 200 images extracted from the MIMIC-CXR database. Uncertainty quantification was performed using the level of confidence in the ETT–carina distance prediction. Segmentation of the ETT was carried out using edge detection and pixel clustering.

**Results:**

The interrater agreement was 0.18 cm for the ETT tip position, 0.58 cm for the carina position, and 0.60 cm for the ETT–carina distance. The mean absolute error of the model on the external test set was 0.51 cm for the ETT tip position prediction, 0.61 cm for the carina position prediction, and 0.89 cm for the ETT–carina distance prediction. The assessment of ETT placement was improved by complementing the human interpretation of chest radiographs with the CarinaNet model.

**Conclusions:**

The CarinaNet model is an efficient and generalizable deep learning algorithm for the automated assessment of ETT placement on ICU chest radiographs. Uncertainty quantification can bring the attention of intensivists to chest radiographs that require an experienced human interpretation. Image segmentation provides intensivists with chest radiographs that are quickly interpretable and allows them to immediately assess the validity of model predictions. The CarinaNet model is ready to be evaluated in clinical studies.

**Graphical Abstract:**

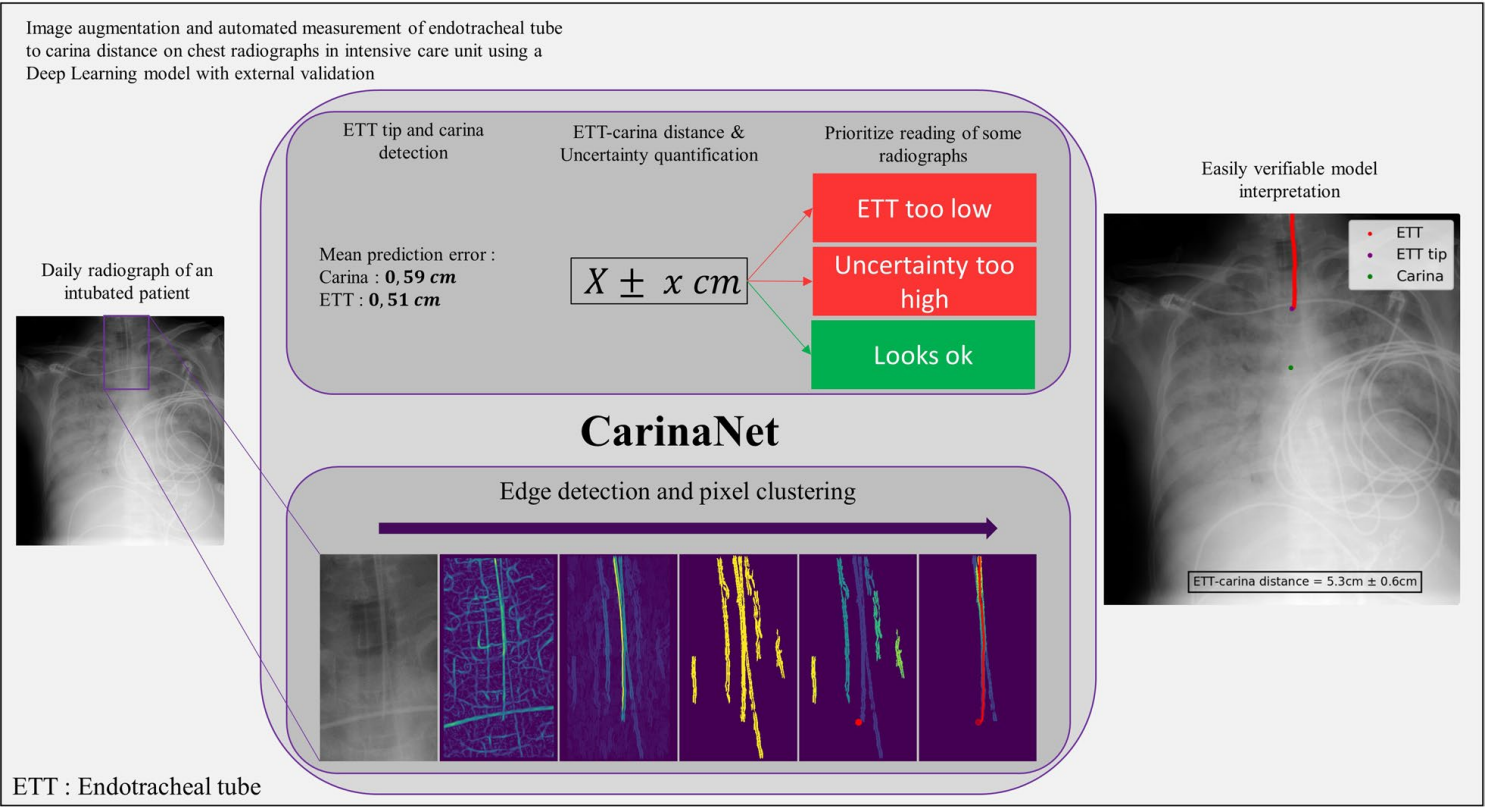

## Background

Chest radiographs in the intensive care unit (ICU) are routinely performed to search for signs of pneumonia and pleural effusion or to check the placement of gastric tubes, central catheters, and endotracheal tubes (ETT) [1–3].

In the ICU, chest radiographs are usually performed with the patient in a semi-sitting or supine position. As a result, they are often of inferior quality to those performed in the radiology department, and their interpretation typically requires substantial time and expertise [4]. In this context, the automated interpretation of chest radiographs using artificial intelligence could help save time and limit human error by focusing the attention of intensivists on malpositioned ETTs. Artificial intelligence algorithms have shown excellent performance in medical imaging, with deep learning algorithms proving especially effective for the interpretation of fundus images [5, 6], brain magnetic resonance imaging [7], cardiovascular imaging [8], and chest radiographs [9]. The good performance of artificial intelligence algorithms for the assessment of ETT placement on chest radiographs has also been demonstrated. Some studies have successfully used template matching and shape recognition of anatomical landmarks for the detection of the ETT tip and carina [10, 11]. Others have shown the effectiveness of deep learning algorithms trained for the segmentation of the ETT [12], the detection of the ETT tip and carina [13], or the prediction of the ETT–carina distance [14]. However, none of these algorithms includes uncertainty quantification, and none combines ETT tip and carina detection with ETT segmentation. This is unfortunate, as uncertainty quantification would increase the reliability of model predictions, while ETT segmentation would improve the interpretability of chest radiographs in real-world use.

The aim of this study was to propose an externally validated deep learning model with uncertainty quantification and image segmentation for the automated assessment of ETT placement on unselected ICU chest radiographs.

## Methods

### Study design

The CarinaNet model was constructed by applying transfer learning to the RetinaNet model using an internal dataset composed exclusively of unselected ICU chest radiographs. This dataset called RadioICU was created especially for this work.

The accuracy of the CarinaNet model in predicting the position of the ETT tip and carina on ICU chest radiographs was externally validated using a dataset extracted from the MIMIC-CXR database, a US multicenter database of chest radiographs [15].

Uncertainty quantification was performed using the level of confidence in the ETT–carina distance prediction. Segmentation of the ETT was carried out using edge detection and pixel clustering. In accordance with the Consort-AI guidelines [16], the model code and weights are available at https://github.com/USM-CHU-FGuyon/CarinaNet .

### Radio ICU dataset

The RadioICU dataset was composed of all consecutive chest radiographs performed in the polyvalent ICU of Reunion Island University Hospital between 01/01/2016 and 12/31/2019. All radiographs were acquired using a Siemens (München, Germany) Mobilett XP Hybrid Analog Imager with Fuji (Tokyo, Japan) radio luminescent memory screens.

Chest radiographs showing non-intubated patients were retained. Those showing intubation through tracheostomy were dropped from the dataset.

Radiographs were converted from their original DICOM format to 8-bit PNG images and the PixelSpacing attribute was extracted for the conversion from pixel to centimeters. The mean resolution was 3.0 ± 0.5 megapixels. The images were anonymized and did not contain burned-in text.

Images were independently annotated by two experienced intensivists (JA or AR), over a predetermined 3-day period. Intensivists were blinded to each other. The annotation of images included the following information:

- A subjective quality score between 0 = *barely readable*; 1 = *hardly visible carina*; 2 = *poor quality*; 3 = *acceptable quality*;
- The pixel coordinates of the ETT tip;
- The pixel coordinates of the carina;
- A classification of the ETT tip relative to the carina : *good*; *too high*; *too low*; *bronchial insertion*

No coordinates were provided when the carina or the ETT tip could not be located. The annotated pixel coordinates were used as ground truth positions of the ETT tip and carina. A 2-cm distance is commonly cited in the literature as a minimal threshold for the positioning of the ETT relative to the carina [2, 17]. Hence, the ETT position was defined as *too low* when the ground truth ETT–carina distance was lower than 2*cm*. The intensivists were asked to classify the *too low* ETTs following their clinical practices, and were informed that the 2-cm threshold would be used as ground truth for the identification of *too low* ETTs. After classification, they annotated precisely the pixel coordinates of the ETT tip and carina.

Among radiographs where both the ETT tip and carina coordinates were provided, 200 were randomly selected. This constituted the internal test. No radiographs were excluded on criteria of quality or readability.

### MIMIC-CXR dataset

The CarinaNet model was externally validated using a dataset extracted from the MIMIC-CXR database, a US multicenter database of chest radiographs. This external test set allowed to evaluate the generalizability of the CarinaNet model [15].

The MIMIC-CXR dataset was created by randomly selecting 200 chest radiographs featuring an ETT from the MIMIC-CXR database. Radiographs were selected without consideration for image quality or medical report content. Unlike the RadioICU dataset, the MIMIC-CXR dataset contained images with burned-in text. The MIMIC-CXR dataset had higher resolution than the RadioICU dataset images.

On a given day ($t_0$), the chest radiographs of the MIMIC-CXR dataset were independently annotated by two experienced intensivists (NA and JA). Ten days later ($t_{10}$), the chest radiographs of the MIMIC-CXR dataset were shuffled and given back to one of the two intensivists (JA) for a second blinded annotation. The annotations at $t_0$ and $t_{10}$ consisted of the pixel coordinates of the ETT tip and carina.

The ground truth positions of the ETT tip and carina were defined by averaging the two annotations made at $t_0$. The interrater agreement was defined as the mean absolute difference between the two annotations made at $t_0$. The intrarater agreement was defined as the mean absolute difference between the annotations made by the same intensivist (JA) at $t_0$ and $t_{10}$. Finally, the ETT position was defined as *too low* when the ground truth ETT–carina distance was lower than 2*cm*.

### The CarinaNet model: a deep learning model for the automated assessment of endotracheal tube placement on chest radiographs

The CarinaNet model was constructed by applying transfer learning to the RetinaNet model, an object detection model developed by Facebook AI Research (New York, USA) [18]. The RetinaNet model has shown state-of-the-art performance on the Common Objects in Context dataset which consists in the detection of specific objects in photographs of everyday life [19]. It has also been used with great success in aerial imagery [20, 21], and medical imaging (computed tomography scans [22], ultrasounds [23], chest radiographs [24], etc.).

**Fig. 1:**
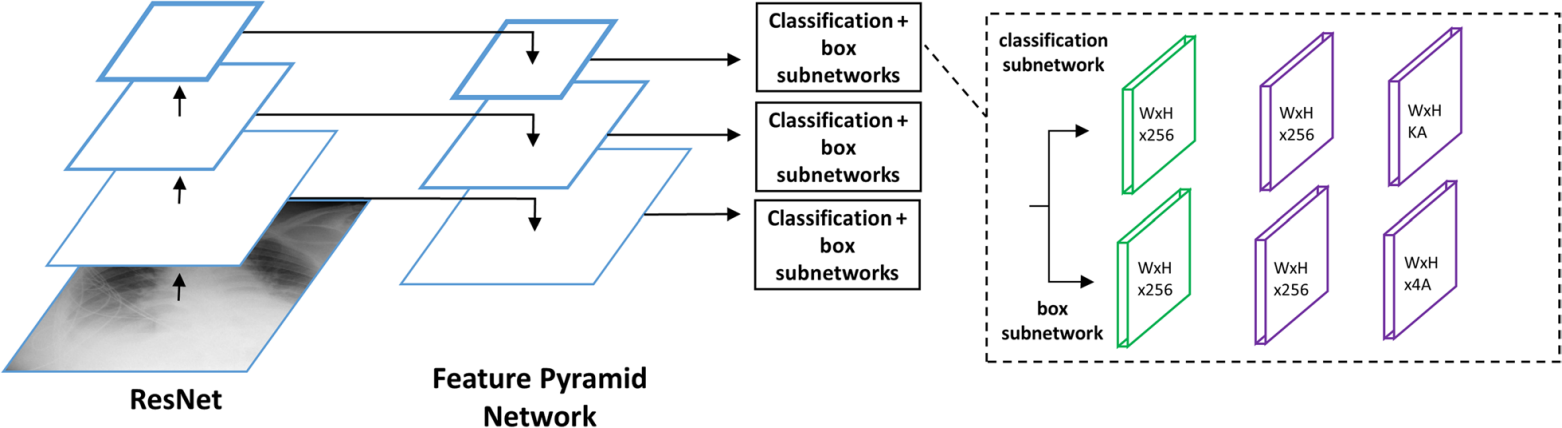
Architecture of the RetinaNet model

The RetinaNet architecture combines a ResNet architecture with a Feature Pyramid Network that outputs a series of feature maps at different spatial scales [25]. These feature maps are fed to two subnetworks: 1) a box subnetwork that builds an anchor box around the object; and 2) a classification subnetwork that classifies the anchor boxes (Fig. 1).

**Fig. 2:**
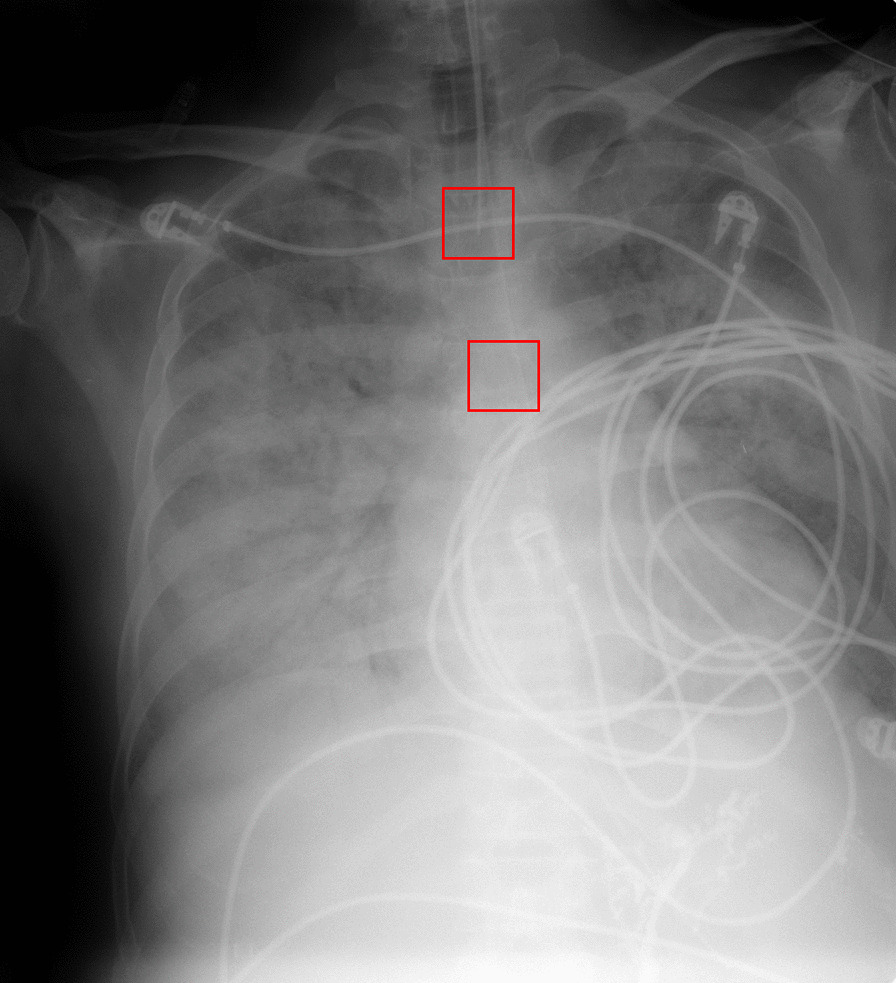
Bounding boxes around the carina and the endotracheal tube tip on a chest radiograph

The CarinaNet model was constructed by training the RetinaNet model on the RadioICU chest radiographs. For this purpose, clinician’s annotations were converted to ($200 \times 200$ pixel) bounding boxes around the pixel coordinates of the ETT tip and carina, as shown in Fig. 2.

For each image, the model output a bounding box for the ETT tip and carina each classified with a confidence score. The position predictions were defined as the center of each bounding box.

### Model performance assessment

We evaluated the model performance by computing the mean absolute error between the ETT tip position prediction, the carina position prediction, the ETT–carina distance prediction and their respective ground truths.

The mean errors between the prediction and ground truth for the ETT tip position, carina position, and ETT–carina distance were computed using a linear mixed model. Correlation between measures was accounted for by using a compound symmetry variance–covariance matrix. The same method was used for assessing the interrater and intrarater mean errors. All statistical tests were performed at a two-tailed type I error of 5%.

### Uncertainty quantification

To increase the reliability of the CarinaNet model, uncertainty quantification was performed using the level of confidence in the ETT–carina distance prediction.

The level of confidence in the ETT–carina distance prediction was defined as the lowest confidence score between the ETT tip position prediction and the carina position prediction. Expression (1) was used to give the uncertainty *U* of the ETT–carina distance prediction from the level of confidence C.

1 $$\begin{aligned} U = \alpha + \beta \ \mathrm{e}^{-\gamma C} \end{aligned}$$

The ETT tip position was classified as follows :

2 $$\begin{aligned} {\left\{ \begin{array}{ll} \text {Good position},&{} \text {if } D_{pred} - U_{pred} \ge 2cm \\ \text {Too low}, &{} \text {if } D_{pred} - U_{pred} < 2cm \end{array}\right. }\text {,} \end{aligned}$$

where $D_{pred}$ is the ETT–carina distance prediction and $U_{pred}$ is the uncertainty of the ETT–carina distance prediction.

The use of uncertainty quantification allowed for a higher sensitivity in the classification of *too low* ETTs. Chest radiographs showing a *bronchial insertion* were classified along with radiographs showing a *too low* position of the ETT tip.

Lastly, the accuracy in classifying the ETT tip position as *good* or *too low* was compared between a clinician, the CarinaNet model, and a coupled reading. In the latter case, the ETT tip position was classified as *too low* when either the model or the clinician had classified it as such.

### Image augmentation

For each chest radiograph, the following operations were performed to identify the full ETT.

First, a 6-cm-wide rectangular region of interest was selected that extended from 1 cm below the prediction to the top of the image. The resulting smaller image contained the entire ETT.

Second, ridge detection was performed by computing the eigenvalues of the hessian matrix of the image [26], knowing that in image processing a ridge denotes a bright curve against a darker background. This operation resulted in an edge map, containing only the brighter and sharper elements of the chest radiograph.

Third, all non-vertical elements were removed from the edge map using vertical filter (3) on the assumption that the ETT was nearly vertical on all chest radiographs. Morphological opening was applied to the edge map for noise reduction. A binary edge map was obtained by setting the first decile pixel values to 1 and the rest to 0. This binary image was treated as a 2D point cloud.

3 $$\begin{aligned} \begin{bmatrix} 1 &{} 0 &{} -1\\ \vdots &{} \vdots &{} \vdots \\ 1 &{} 0 &{} -1 \end{bmatrix}_{10\times 3} \end{aligned}$$

Fourth, clustering of the binary edge map was performed using DBSCAN (Density Based Spatial Clustering of Applications with Noise) [27]. This algorithm clusters point clouds based on the density of neighboring points.

**Fig. 3:**
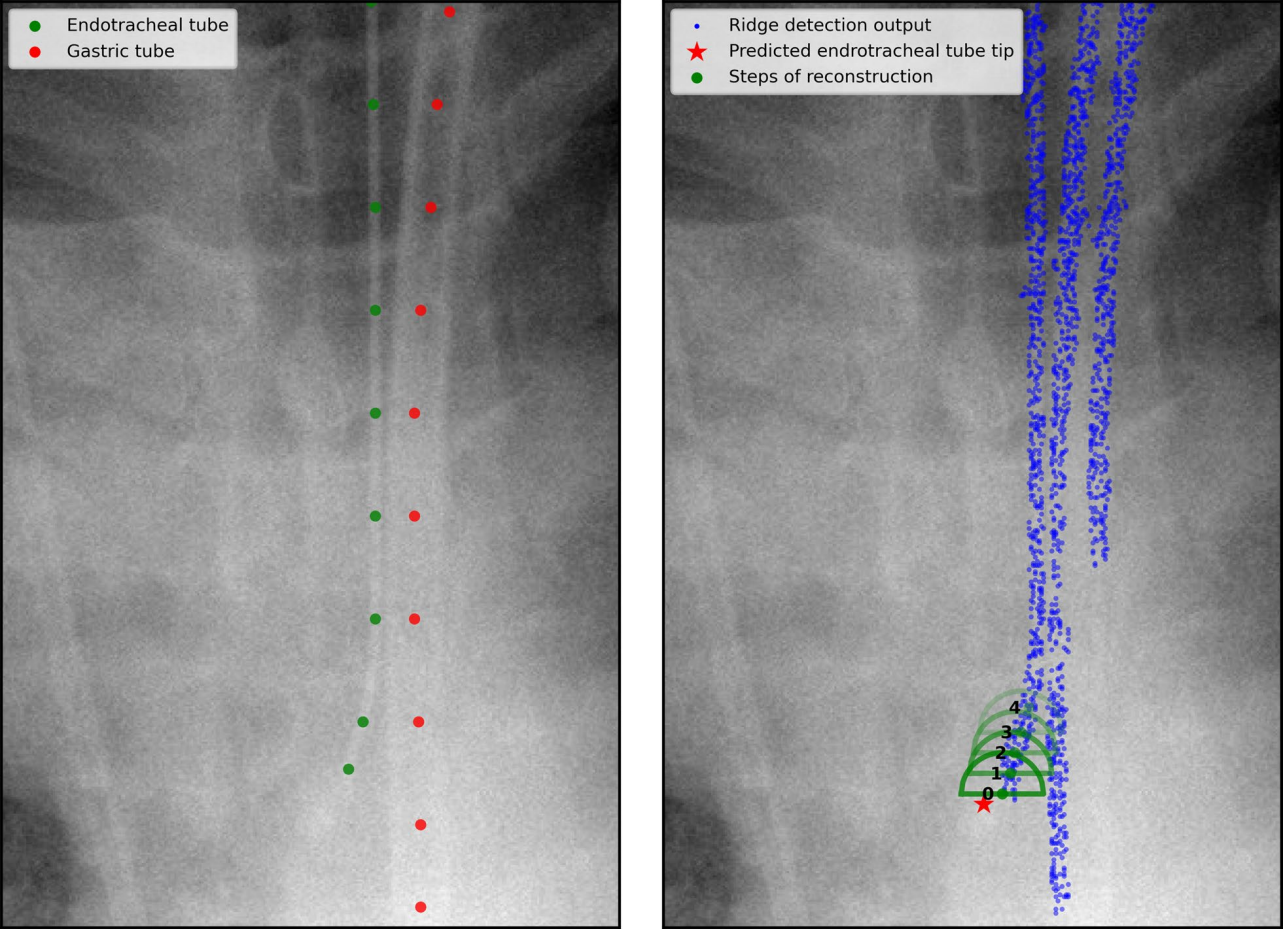
Illustration of the cluster thinning algorithm

Fifth, the ETT was segmented using a cluster thinning algorithm developed specifically for this study and is illustrated in Fig. 3.

This algorithm selected the cluster located closest to the CarinaNet prediction of the ETT tip. It started with the point of the cluster located closest the CarinaNet prediction and then iteratively propagated upwards along the cluster.

Finally, the model output a list of points following a single ridge, which constituted the ETT segmentation.

## Results

### Annotation of chest radiographs

#### RadioICU dataset

The RadioICU dataset was composed of 1,890 separate radiographs, 1,357 of which had annotations for both the ETT and carina. The clinicians rated 12.9% of the radiographs as *acceptable quality*, 70.1% as *poor quality*, 15.6% as *hardly visible carina*, and 1.3% as *barely readable*.

**Fig. 4:**
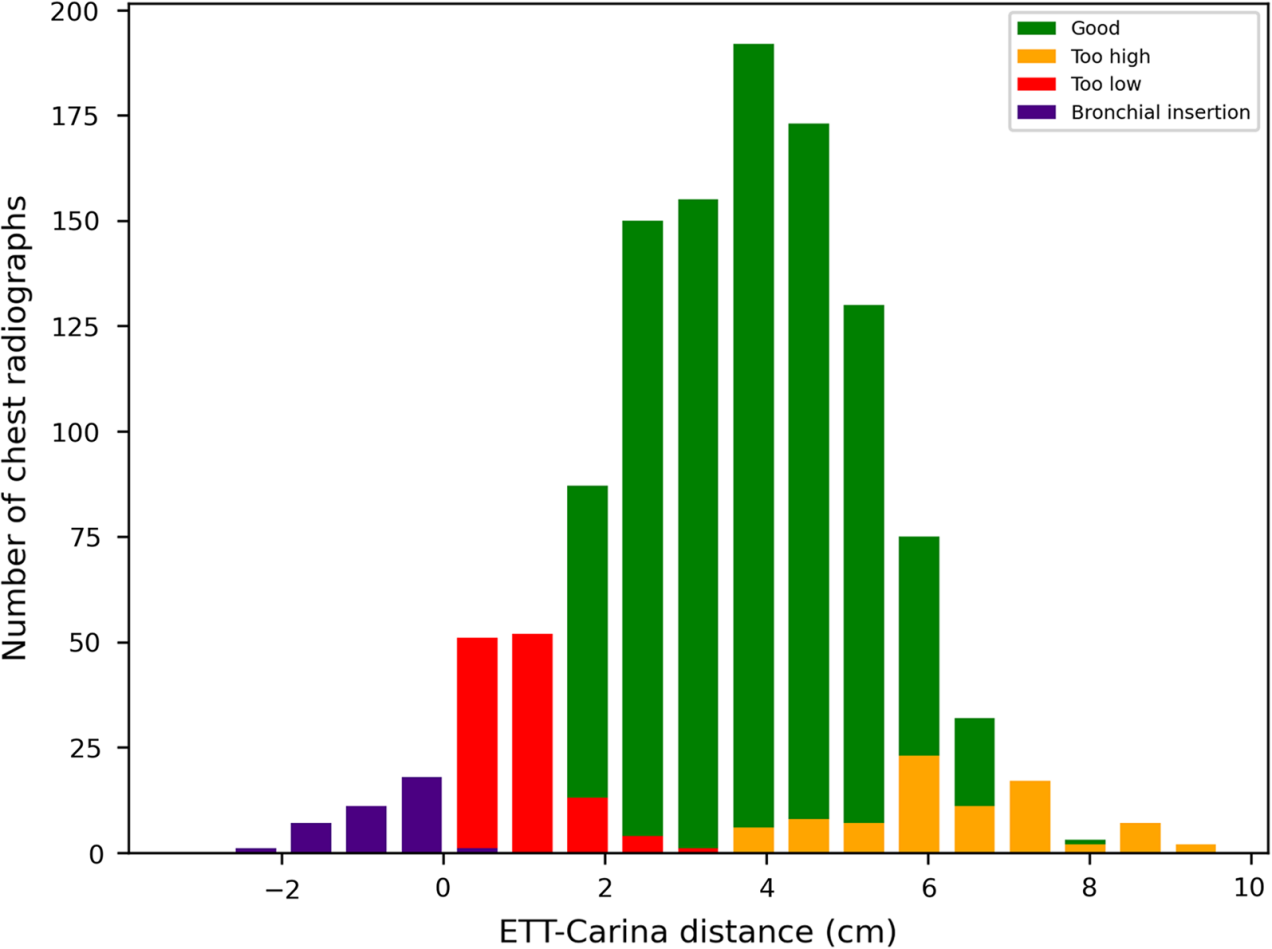
Number of chest radiographs per classification of the endotracheal tube tip position as a function of the annotated endotracheal tube–carina distance

Clinicians generally classified the position of the ETT tip as *too low* when the ETT–carina distance was less than 2 cm (Fig. 4).

#### Mimic-CXR dataset

The interrater agreement and intrarater agreement obtained from the annotation of 200 chest radiographs are given in Table 1. The intrarater agreement was slightly lower than the interrater agreement. The latter was used as the reference for human accuracy in interpreting chest radiographs in clinical practice. Table 1 indicates that the readings of the ETT tip and carina positions from the same intensivist (JA) at a 10-day interval were not statistically different, whereas the readings from two separate intensivists (NA and JA) were statistically different.

### Predictive performance of the CarinaNet model

The mean absolute error of the CarinaNet model for the ETT tip position prediction, the carina position prediction, and the ETT–carina distance prediction performed on the internal and external test sets are shown in Table 1. The mean absolute error on radiographs from the external test set where the level of confidence was superior to 0.5 was also computed. The CarinaNet mean error for the prediction of the ETT tip position, carina position, and ETT–carina distance indicated that the model predictions were not statistically different from the ground truth positions.

**Table 1:** Model performance, interrater and intrarater agreements for the position of the endotracheal tube (ETT) tip, carina, and ETT–carina distance

Element	Test set	Measure	Sample size	Mean absolute error (cm)${}^\mathrm{a}$	Mean error (cm)${}^\mathrm{b}$	*p* value${}^\mathrm{c}$
ETT tip	Internal	CarinaNet prediction	200	0.59	− 0.11	0.23
				(1.03)	[− 0.28, 0.07]	
	External	CarinaNet prediction	200	0.51	0.064	0.41
				(1.08)	[− 0.09, 0.22]	
		CarinaNet prediction with high confidence	101	0.49	0.066	0.09
				(0.61)	[− 0.01, 0.14]	
		Interrater agreement	200	0.18	− 0.12	< 0.0001
				(0.30)	[− 0.17, − 0.08]	
		Intrarater agreement	200	0.15	− 0.015	0.53
				(0.33)	[− 0.061, 0.031]	
Carina	Internal	CarinaNet prediction	200	0.60	0.059	0.42
				(1.25)	[− 0.08, 0.20]	
	External	CarinaNet prediction	200	0.61	0.019	0.76
				(0.90)	[− 0.11, 0.15]	
		CarinaNet prediction with high confidence	101	0.28	0.13	0.04
				(0.61)	[0.01, 0.25]	
		Interrater agreement	200	0.58	− 0.30	< 0.0001
				(0.88)	[− 0.43, − 0.18]	
		Intrarater agreement	200	0.48	0.056	0.34
				(0.84)	[− 0.06, 0.17]	
ETT–carina distance	Internal	CarinaNet prediction	200	0.90	− 0.16	0.14
				(1.55)	[− 0.38, 0.05]	
	External	CarinaNet prediction	200	0.89	0.045	0.66
				(1.49)	[− 0.15, 0.24]	
		CarinaNet prediction with high confidence	101	0.59	− 0.061	0.41
				(0.74)	[− 0.21, 0.08]	
		Interrater agreement	200	0.60	0.17	0.009
				(0.93)	[0.05, 0.30]	
		Intrarater agreement	200	0.54	− 0.071	0.26
				(0.89)	[− 0.20, 0.05]	

$^\mathrm{a}$The standard deviation is shown in parentheses

$^\mathrm{b}$The interval of confidence shown in brackets. The mean error and interval of confidence were obtained using a linear mixed model that accounted for intraclass correlation

$^\mathrm{c}$The *p* value refers to the mean error obtained using the a linear mixed model

Table 2 shows the model’s performance on the RadioICU dataset for each radiograph quality. The best predictive performance was obtained with the more frequent radiograph qualities (*poor quality*, *hardly visible carina*) rather than with the *acceptable quality* radiographs.

**Table 2:** Mean absolute error on all images of the RadioICU dataset where coordinates were provided for both the endotracheal tube and carina. ETT : endotracheal tube

	ETT tip	Carina	ETT–carina distance
*Barely readable*, n= 14	1.98 ± 3.22	0.49 ± 0.65	2.36 ± 3.57
*Hardly visible carina*, n= 193	0.36 ± 0.83	0.49 ± 0.80	0.61 ± 1.03
*Poor quality*, n = 1100	0.43 ± 1.13	0.44 ± 0.96	0.62 ± 1.25
*Acceptable quality*, n= 50	0.49 ± 1.32	0.54 ± 1.38	0.73 ± 1.53

The results in this table are formatted as [mean ± standard deviation].

### Uncertainty quantification

**Fig. 5:**
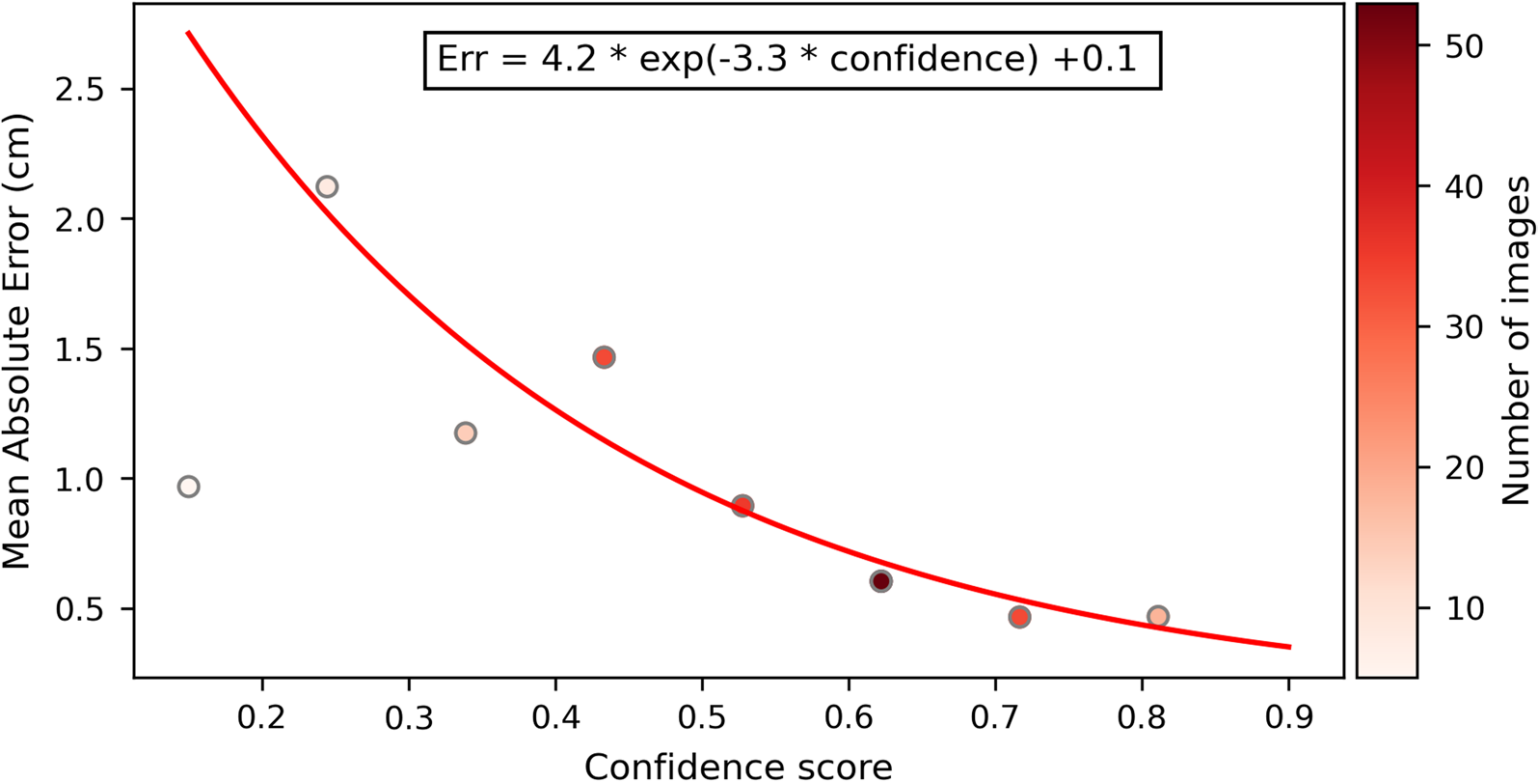
Exponential fit of the absolute error for the endotracheal tube–carina distance prediction as a function of the level of confidence in this prediction. The predictions were grouped into 8 bins based on the level of confidence and the mean absolute error was computed for each bin

Figure 5 indicates an exponential decrease in the absolute error of the ETT–carina distance prediction with the increase in the level of confidence. This justified the use of equation (1).

Based on the exponential fit shown in Fig. 5, the uncertainty quantification of the CarinaNet model was defined as:

4 $$\begin{aligned} U_{pred} = {0.1} + {4.2}\ \mathrm{e}^{{-3.3}C} \end{aligned}$$

where $U_{pred}$ is the uncertainty of the ETT–carina distance prediction and *C* is the level of confidence in the ETT–carina distance prediction.

**Fig. 6:**
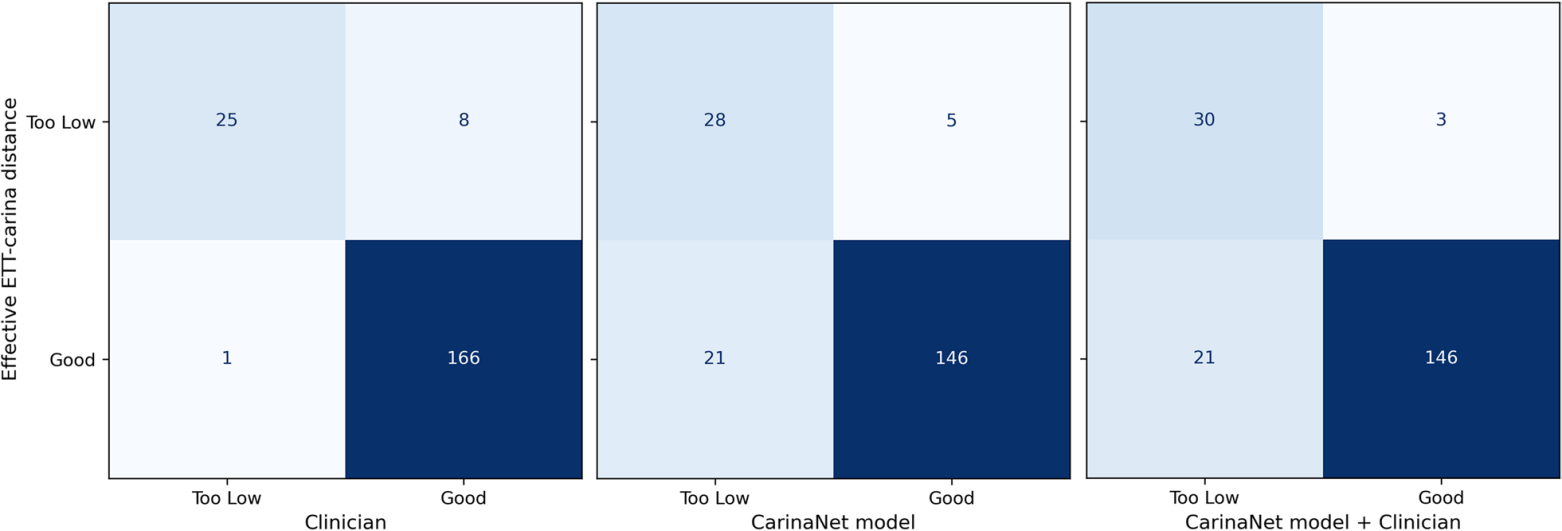
Confusion matrices for the classification of the endotracheal tube tip position by a clinician, the CarinaNet model, and a coupled reading

Confusion matrices for the classification of the ETT tip position by a clinician, the CarinaNet model, and a coupled reading are shown in Fig. 6. The coupled reading classified the ETT tip as *too low*, when either the CarinaNet model or the clinician classified it as such. In each matrix, the classification of the ETT tip position was compared to a measure of the effective ETT–carina distance. As Fig. 6 indicates, 8 chest radiographs were wrongly classified as showing a well-positioned ETT tip when the classification was performed by a clinician. This number fell to 3 when the classification was performed by a clinician together with the CarinaNet model.

The sensitivity and specificity of the classification of *too low* ETT tips by a clinician, the CarinaNet model, and a coupled reading are shown in Table 3. The lower specificity of the CarinaNet model was expected when using classification criterium (2), but induced a better sensitivity in the context of a combination with the human interpretation.

**Table 3:** Sensitivity and specificity of the classification of low endotracheal tip location by a clinician, the CarinaNet model, and a coupled reading

	Sensitivity	Specificity
Clinician	0.76 [0.60, 0.91]	0.99 [0.98, 1.0]
CarinaNet Model	0.85 [0.73, 0.97]	0.87 [0.82, 0.92]
Clinician + CarinaNet Model	0.91 [0.80, 1.0]	0.87 [0.82, 0.92]

The results in this table are formatted as [value [95% confidence interval]].

### Image segmentation

**Fig. 7:**
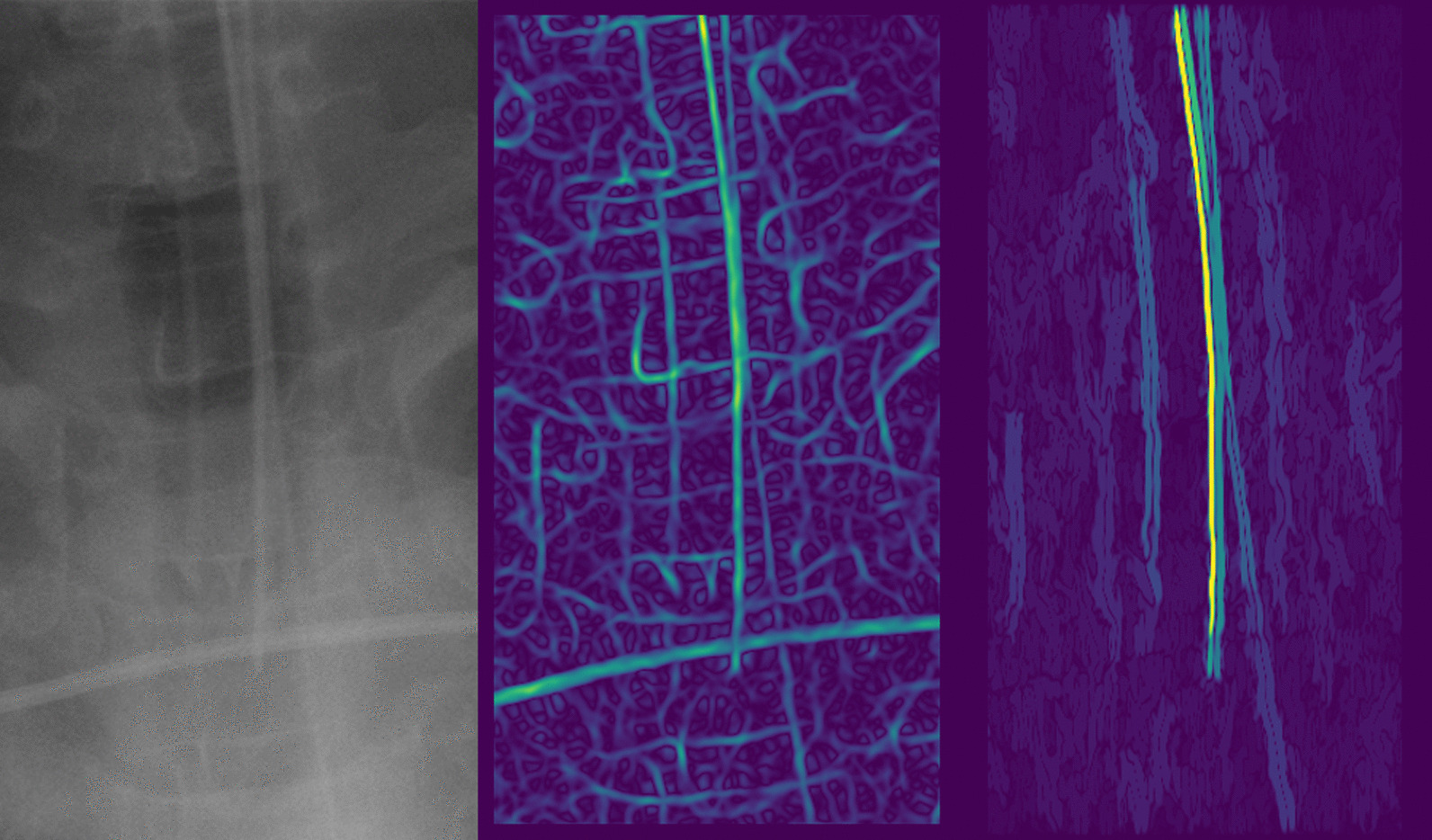
Edge detection on the region of interest

**Fig. 8:**
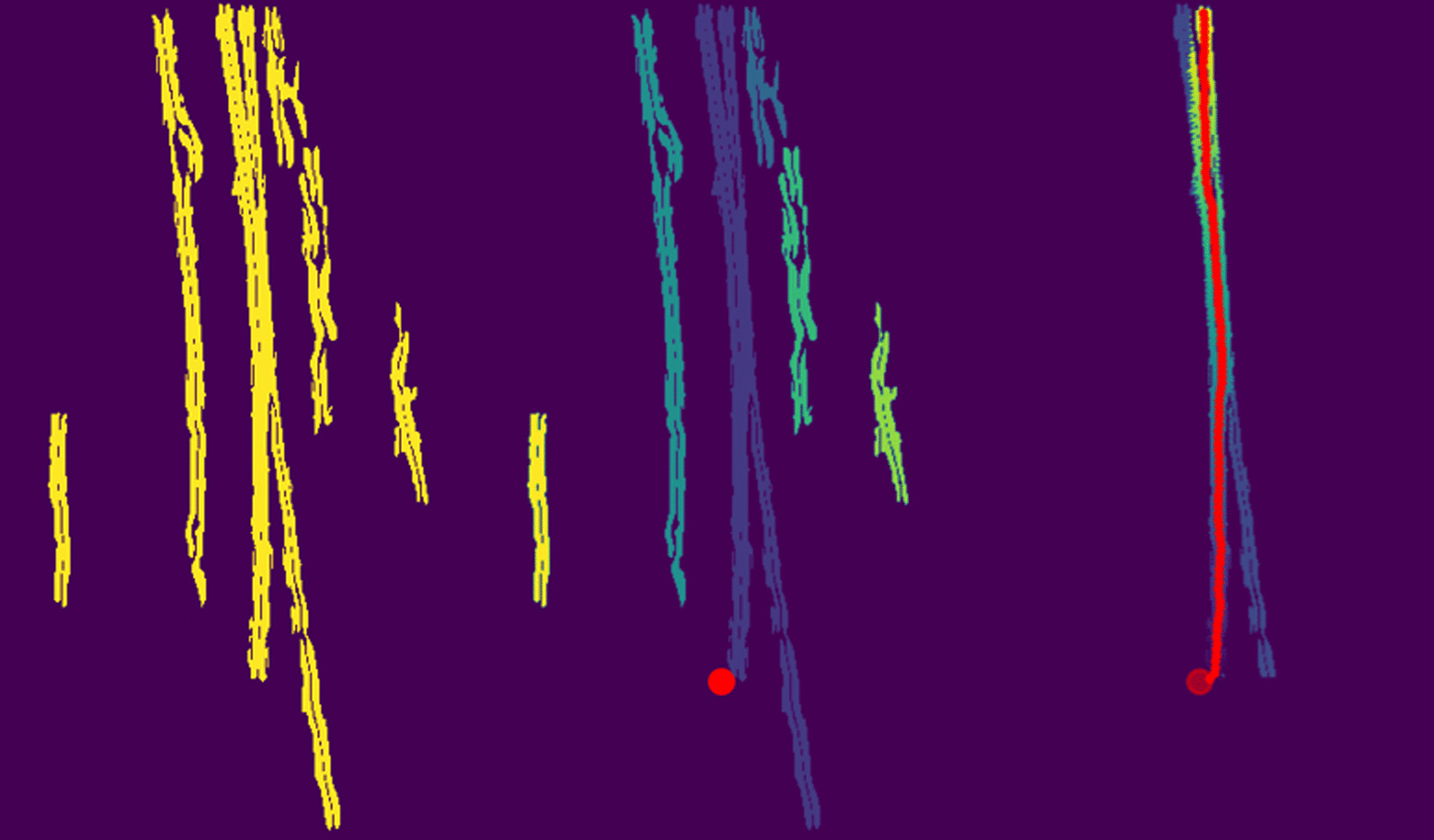
Clustering and cluster thinning on the binary edge map

The successive steps of the image segmentation performed on a chest radiograph of the RadioICU dataset are shown in Figs. 7 and 8 .

First, a region of interest centered on the ETT was obtained thanks to the good accuracy of the ETT tip position prediction (Fig. 7).

Second, the ridge detection algorithm was applied to the chest radiograph. The resulting edge map clearly highlighted the ETT. However, other elements of the region of interest were also highlighted, in particular the horizontal cable at the bottom of the image and the small artifacts on either side of the ETT (Fig. 7).

Third, all non-vertical elements and smaller artifacts were removed from the original image by applying the vertical filter (3) to the edge map and then performing morphological opening. The horizontal cable and the smaller edges totally disappeared, leaving the entire ETT clearly highlighted (Fig. 7). After binarization of the filtered edge map, the only remaining elements were the ETT, the gastric tube, and certain artifacts (Fig. 8).

Fourth, non-overlapping objects were separated by applying the DBSCAN algorithm to the binary edge map. The artifacts located on either side of the ETT were classified into separate clusters. As the gastric tube considerably overlapped with the ETT on the original chest radiograph, the DBSCAN algorithm generated a single cluster containing both objects (Fig. 8).

Fifth, the cluster thinning algorithm was applied to the binary edge map. This operation highlighted the ETT but not the gastric tube (Fig. 8).

**Fig. 9:**
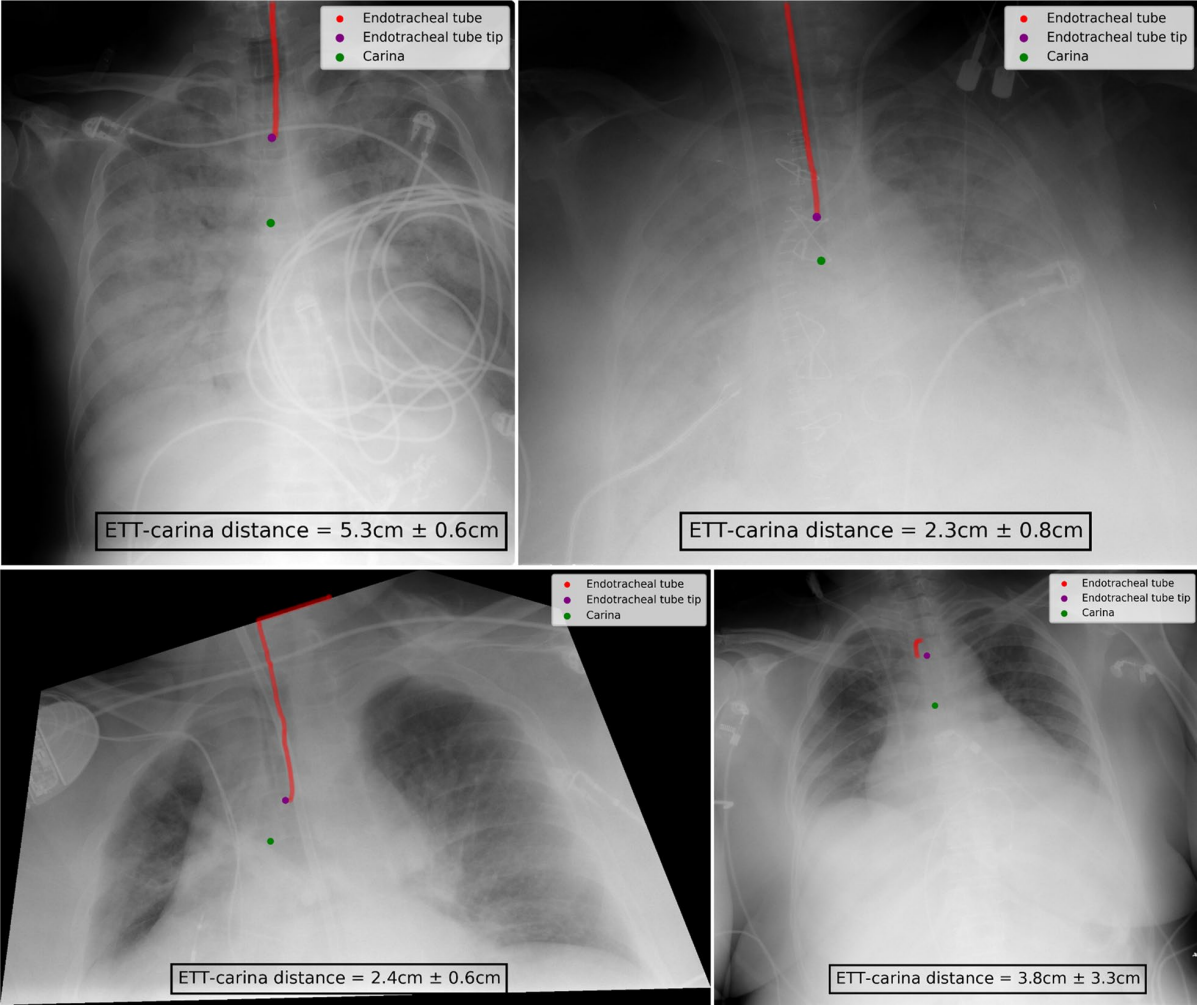
Example outputs of the CarinaNet model. Top-left and top-right chest radiograph were, respectively, classified as having *acceptable quality* and a *hardly visible carina*. The endotracheal tube tip and carina were accurately detected and the model uncertainty was inferior to 1 cm, indicating that the model result could be trusted. The segmentation was successful for both these radiographs. Bottom-left radiograph had a very unusual angle, this disrupted the segmentation step. However the endotracheal tube was entirely segmented and the endotracheal tube tip and carina were accurately detected. Bottom-right radiograph featured no endotracheal tube. The CarinaNet model properly detected the carina and output an endotracheal tube tip position but with an uncertainty of 3.3 cm indicating that the result could not be trusted. Note that the segmentation step also failed

The output of the cluster thinning algorithm constituted the ETT segmentation. The segmentation obtained from the successive operations shown in Figs. 7 and 8 is visible on the top-left radiograph in Fig. 9. As Fig. 9 indicates, the CarinaNet model predicted the ETT–carina distance with very low uncertainty. Bottom-left radiograph of Fig. 9 shows the segmentation of the ETT on a tilted radiograph. On this radiograph, the ETT was entirely segmented, but the segmentation also selected the border of the image.

## Discussion

This is the first study to propose an externally validated deep learning algorithm with uncertainty quantification and image segmentation for the automated assessment of ETT placement on unselected ICU chest radiographs. The main benefit of the CarinaNet model is that it can help intensivists prioritize chest radiographs that have been pre-classified as featuring a too low ETT. Uncertainty quantification is also a major security contribution in that it can bring the attention of intensivists to chest radiographs that require an experienced human interpretation. Lastly, the usability of the CarinaNet model is reinforced by image segmentation, which provides intensivists with chest radiographs that are quickly interpretable and allows them to immediately assess the validity of model predictions.

Little difference was observed between the internal validation of the CarinaNet model and its external validation. Indeed, the mean absolute error of the model for the ETT tip position, carina position and ETT–carina distance was 0.51 cm, 0.61 cm, 0.89 cm on the external test set compared to 0.59 cm, 0.60 cm, 0.90 cm on the internal test set, respectively.

This good generalization of the CarinaNet model indicates that it is not disturbed by unfamiliar elements on chest radiographs. More generally, the good predictive performance of the model suggests that it is usable in clinical practice.

Previous studies have shown the interest of using deep learning algorithms for the automated interpretation of ICU chest radiographs. In a study evaluating the InceptionV3 model, external validation on 100 images yielded an error of 0.63 ± 0.55 cm for the ETT–carina distance prediction [14]. This suggests a better performance than the CarinaNet model; however, the model has low interpretability as it did not explicitly detect the ETT tip and carina. Another study evaluated a cascaded convolutional neural network model for its accuracy in predicting the ETT tip or carina position on ICU chest radiographs from the MIMIC-CXR database [13]. The prediction error of this model on the internal test set was 0.82, 0.64 and 0.86 cm for the ETT tip position, carina position, and ETT–carina distance, respectively [13]. While these findings are similar to ours, it should be noted that this study proposed no external validation. Moreover, in both these studies the models were evaluated on radiographs for which the ETT–carina distance was already specified in the medical report. This likely induced a selection bias.

The CarinaNet model was able to identify *too low* ETT tip positions with a sensitivity of 0.85 and a specificity of 0.87. The sensitivity and specificity of the classification performed by a clinician were 0.76 and 0.99, respectively, while those of the classification performed by a clinician coupled with the CarinaNet model were 0.91 and 0.87, respectively. This increase in sensitivity corresponds to increased security compared to current clinical practice. The lower specificity of the CarinaNet model indicates that pre-classification may prioritize the clinician’s reading of some chest radiographs showing a well-positioned ETT. Thus, this pre-classification remains a strict improvement from current clinical practice. Our findings support studies that recommend using artificial intelligence as a complement to human tasks rather than as a substitute [28].

There was a considerable difference between the prediction error of the CarinaNet model and the interrater agreement for the ETT tip position. This can be explained by the fact that the ETT was designed to be radiopaque and easily identifiable by the human eye. By contrast, the prediction error was very close to the interrater agreement for the carina position. The model performance even surpassed the interrater agreement on the carina detection when considering predictions where the level of confidence was superior to 0.5, which was half of the dataset. These findings suggest that deep learning algorithms have different perceptive qualities than the human eye and can therefore be used to complement the human interpretation of chest radiographs.

Additionally, the model predictions were not statistically different from the ground truth for the ETT tip and carina positions. By contrast, the readings of the ETT tip and carina positions by two experienced intensivists were statistically different. This demonstrates that our CarinaNet model could improve the current clinical practices by reducing the impact of the inter-intensivist variability for the reading of the routine chest radiographs.

Interestingly, the lowest prediction errors of the CarinaNet model were obtained not for chest radiographs classified by intensivists as being of *acceptable quality*, but for those of lower quality, which were the most frequent. This shows that deep learning models trained with hard examples have good adaptability to challenging tasks. The CarinaNet model yielded the highest prediction errors for images taken from a very unusual angle (see Fig. 9) or featuring opaque lungs. This problem could be addressed by increasing the proportion of tilted images or images of opaque lungs in the training set.

Moreover, we designed our model to be used solely on chest radiographs of intubated patients because we believe the pre-interpretation should only be made when clinically relevant. As a consequence, our model did not explicitly check for the presence of an endotracheal tube. However, our built-in uncertainty quantification acted as a safeguard by indicating that the result could not be trusted when the uncertainty was too high (bottom-right radiograph of Fig. 9). This demonstrates that integrated uncertainty quantification is a major robustness contribution for the clinical application of our model.

Our study has some limitations. First, since the images of the training set were non-redundantly annotated, the prediction error of the model was necessarily higher than the interrater agreement for the carina position. Second, few of the test set images showed a malpositioned ETT, which induced imprecise sensitivity and specificity values for the model’s classification. This problem could be overcome by using test sets with a larger proportion of images showing a malpositioned ETT. Third, the coupled reading was simulated using the model’s classification and the clinician’s blinded annotation. Our results thus underestimate the benefit of using the CarinaNet model as the clinician’s reading should be evaluated with the knowledge of the model’s classification as well as the augmented image. Finally, the use of the 2-cm criterion could be debated as no study provided evidence for the superiority of this threshold against another. However, our model is adaptable to any given threshold if recommendations change in the future.

In the future, the predictive performance of the CarinaNet model could be increased by using a training set with a larger proportion of images showing a malpositioned ETT. Data augmentation in training or at test time could also improve the performance of the model. Lastly, studies should be performed to validate the applicability of the model in clinical practice.

## Conclusion

In conclusion, the CarinaNet model is an efficient and generalizable deep learning algorithm for the automated assessment of ETT placement on ICU chest radiographs. This model can help intensivists prioritize chest radiographs that have been pre-classified as featuring a malpositioned ETT. Moreover, uncertainty quantification can bring the attention of intensivists to chest radiographs that require an experienced human interpretation. Finally, image segmentation provides intensivists with chest radiographs that are quickly interpretable and allows them to immediately assess the validity of model predictions. The CarinaNet model is ready to be evaluated in clinical trials.

## Data Availability

The RadioICU dataset—used for training and internal validation—is not publicly available. The MIMIC-CXR dataset—used for external validation—is publicly available at https://physionet.org/content/mimic-cxr/2.0.0/. All the codes and model weights needed to reproduce the results are available at https://github.com/USM-CHU-FGuyon/CarinaNet.

## Abbreviations

ICU: Intensive care unit
ETT: Endotracheal tube
